# Intestinal Transplantation in a Country Without Home Parenteral Nutrition: The Largest Report from the Middle East

**DOI:** 10.5152/tjg.2022.21708

**Published:** 2022-09-01

**Authors:** Hamed Nikoupour, Mohammad Bagher Khosravi, Pooya Vatankhah, Mojtaba Shafiekhani, Alireza Shamsaeefar, Peyman Arasteh, Mohammad Hossein Anbardar, Mohammad Hossein Eghbal, Mohammad Ali Sahmeddini, Fatemeh Khalili, Mohammad Firoozifar, Samaneh Ghazanfar Tehran, Saman Nikeghbalian

**Affiliations:** 1 Shiraz Transplant Center, Abu Ali Sina Hospital, Shiraz University of Medical Sciences, Shiraz, Iran; 2 Anesthesiology and Critical Care Research Center, Shiraz University of Medical Sciences, Shiraz, Iran; 3 Shiraz Transplant Research Center, Shiraz University of Medical Sciences, Shiraz, Iran; 4 Department of Clinical Pharmacy, Shiraz University of Medical Sciences Faculty of Pharmacy, Shiraz, Iran; 5 Department of Pathology, Abu Ali Sina Hospital, Shiraz University of Medical Sciences, Shiraz, Iran; 6 Department of Anesthesiology, Anesthesiology Research Center, Guilan University of Medical Sciences Alzahra Hospital, Rasht, Iran

**Keywords:** Anesthesia, autologous gastrointestinal reconstruction, fluid therapy, intestine, parenteral nutrition, transplantation

## Abstract

**Background:**

Many regions of the world, especially middle- and low-income countries, lack facilities for home parenteral nutrition and thus cannot follow existing guidelines for intestinal transplantation. Herein, we report our experiences with treatment protocols, intraoperative management, and early postoperative outcomes among patients undergoing either isolated intestinal transplantation or multivisceral transplantation in our center.

**Methods:**

During a 1-year period from March 2019 to March 2020, a total of 9 intestinal transplantations including 6 isolated intestinal transplantations and 3 multivisceral transplantations were performed in our center. We reported on donor selection strategies, surgical treatment, anesthesiology care and protocols for total parenteral nutrition, immunosuppression regimen, and pathology evaluation.

**Results:**

Mean (standard deviation) age of patients was 37.5 ± 12.5 years. The majority of patients were females (7/9). The median (interquartile range) waiting time for patients from diagnosis to transplantation was 79 (34, 164) days. Our 7-day survey of the amount of fluid therapy after transplantation revealed that the greatest need for fluid therapy was seen on the second postoperative day. After transplantation, 2 patients showed a total of 3 episodes of severe rejection, 1 of which was antibody-mediated. The 1-year survival was 66.6% and the 2-year survival was 44.5% in our study population. The median (interquartile range) time to death was 157 (26.5, 382) days. The most common cause of death was sepsis in our series (3/5).

**Conclusion:**

Acceptable outcomes can be obtained with intestinal transplantation in countries without home parenteral nutrition by application of specific treatment protocols.

## Main Points

- In our center, like many regions, facilities for home parenteral nutrition (HPN) do not exist.
- During a 1-year period, a total of 9 patients underwent intestinal transplantation in our center in the Middle East.
- All our transplantations were done among adult patients and our 1-year survival rates (66.6%) were similar to that of other regions of the world among adult patients.
- Our treatment protocols can be applied in regions without HPN with acceptable outcomes.

## Introduction

Intestinal failure (IF) is characterized by reduced gastrointestinal absorption of necessary nutrients to maintain and fulfill the body’s nutritional demands which eventually leads to parenteral nutrition (PN)^1^ and is estimated to occur among every 50 individuals per million population.^2^ The optimum and endpoint treatment for the condition is intestinal transplantation (ITx). This is a complicated procedure that requires a multidisciplinary team and is only performed in a few transplant centers in the world.^3^ Many regions of the world, especially middle- and low-income countries, lack facilities for home parenteral nutrition (HPN)^4^ and thus cannot follow existing guidelines for ITx.

During a 1-year period from March 2019 to March 2020, a total of 9 ITxs including 6 isolated ITxs and 3 multivisceral transplantations (MVTx) were performed in our center. In this study, we aim to report our experiences with treatment protocols, intraoperative management, and early postoperative outcomes among patients undergoing either isolated ITx or MVTx in our center.

## MATERIALS AND METHODS

### Surgical Management of Intestinal Transplantation

#### Treatment Protocol

All patients with IF are referred to the intestinal rehabilitation unit (IRU). Following hospitalization and stabilization of their conditions, the patients will be visited by a multidisciplinary team consisting of transplant surgeons, clinical pharmacologists, psychiatrists, infection specialists, and trained nurses.

A large number of patients undergo autologous gastrointestinal reconstruction (AGIR) and consequently become off-PN. A large number of patients, either before or after receiving AGIR (based on their condition) are scheduled for ITx. Our indications for ITx are shown in Supplement File 1.

Up to October 2020, a total of 107 patients with IF have been treated in our center, among which 60 patients have undergone AGIR. Overall, 24 patients have become candidates for ITx and 5 died before their scheduled transplantation. Considering the lack of facilities for HPN, all the patients admitted to our IRU receive PN. In patients with enterocutaneous fistulas or those with ostomas, initially, GI continuity is established, after which patients are referred for transplantation. The most common cause of IF in our population is short bowel syndrome (SBS) due to mesenteric ischemia.^5^

#### Work-Up

During hospitalization, human leukocyte antigen (HLA) typing, flow cytometry Panel-Reactive Antibody (PRA) complete lab tests, Doppler ultrasonography, magnetic resonance imaging, and 2 times per week pan cultures were performed for the patients.

After collecting the laboratory results, with negative cultures for infections, the patients were prepared for ITx when appropriate donors were found.

The donor criteria were as follows: identical blood groups, age <40 years, body mass index < 25 kg/m^2^, being on low-dose inotrope, hospitalization period of less than 5 days, and living in the same city as the recipient.

All the transplantations were done from deceased donors. The recovery process was mediated by the surgeons in our own center.

#### Preparation and Organ Procurement

Preparing the donor includes stabilizing the condition of the patient, administering appropriate antibiotics, and administering 200 cc of povidone-iodine the night before harvesting organs. HLA of all the patients is checked in less than 6 hours. In all of the harvests, cannulation is done through the right iliac artery, given that all the organ implantations are done in a single center, the aorta conduit is specified for the bowel, and the renal arteries are left on the aorta using a 5-mm stump. Harvesting of the pancreas and kidney is done simultaneously, the pancreas is separated from the intestine in the back table, and superior mesenteric vein branches are ligated separately one by one. With regard to the graft in MVTx, both kidneys and the rest of the organs from the stomach to the terminal ileum and the stump of the aorta are harvested. The right colon is not used in the graft, and University of Wisconsin solution is used for cleaning of organs.

Simultaneous to the procurement, surgery on the recipient is done by another surgical team. With regard to the surgery on the recipient, wide dissection is done because of multiple previous operations. Among patients with diffuse porto-mesenteric thrombosis, initially, the superior mesenteric artery of the celiac is clamped and then dissection is continued for extraction of organs. After surgery, all the patients are transferred to the intensive transplantation unit (ITU).

### Anesthesia Preoperative Assessment

Patients underwent comprehensive preoperative assessment (specifics mentioned in Table 1).

### Anesthesia Intraoperative Care and Management

#### Monitoring and Fluid Therapy

Details on this have been mentioned in Supplement File 2.

*Acid-Base Balance and Blood Loss and Coagulation*

Details on these have been provided in Supplement File 2.

### Postoperative Anesthetic Management

#### Extubation

Patients are sedated using standard methods in the ITU. Both basic monitoring (including heart rate [HR], non-invasive blood pressure [NIBP], electrocardiogram [ECG], and pulse oximetry) and advanced monitoring (including an arterial line for IBP, repeated arterial blood gas (ABG) sampling, and a central venous catheter in order to measure central venous pressure (CVP) and fluid therapy) are routinely applied for all patients. Based on patients’ hemodynamic conditions and postoperative laboratory values, the decision is made either to continue the intubation or to taper the sedation and subsequent extubation.

#### Fluid Therapy and Total Parenteral Nutrition Management

Fluid therapy in the ITU is goal-directed. The aim is to maintain CVP of 5-8 cm H_2_O, MAP ≥ 60 mm Hg, and urine output of ≥0.5 cm^3^/kg per hour. Inotropes are used if necessary in selected cases. Fluid therapy is based on both maintenance and replacement fluid of ileostomy drainage. For maintenance fluid, 1000-1500 cm^3^ (1-1.5 mL/kg/h) of dextrose saline is given during a 24-hour period. Fluid loss from the ileostomy is also totally replaced with balanced electrolyte solutions (Ringer’s lactate or Hartmann’s solution). Ringer’s lactate improves acid–base imbalance by decreasing metabolic acidosis compared to normal saline 0.9% solutions.^6^ Furthermore, albumin is administered until it reaches the level of >3 g/dL.

In case of hemodynamic stability, TPN will be resumed similar to the preoperative protocol within hours after entering the ITU.

#### Acid–Base Balance

During the patient’s ITU hospitalization, acid–base disturbances are measured by ABG every 8 hours until the patient is extubated, after which it is measured on a daily basis. In cases of hemodynamic instability, electrolyte imbalance, or surgical complications, complimentary ABGs are drawn and acid–base disturbances are corrected accordingly.

#### Blood Loss and Coagulopathy

During the ITU course of hospitalization, the goal is to maintain serum hemoglobin levels of 9-10 g/L and when needed packed cells are infused. In cases of other coagulopathies, appropriate blood products are used accordingly.

### Medication, Total Parenteral Nutrition, and Biopsy

#### Total Parenteral Nutrition Protocol

All the patients are first evaluated by a transplant surgeon. Then, patients are referred to a clinical pharmacist to start TPN.

Initially, any electrolyte imbalance is corrected, and in cases, where the patient is not able to tolerate oral feeding or has an ultra-short or SBS, TPN is started as follows: total energy expenditure of 25-30 kcal/kg per day according to anthropometric measurements, laboratory data, and scientific guidelines.^7,8^ Further details have been provided in Supplement File 2.

#### Total Parenteral Nutrition After Intestinal Transplantation

After ITx or MVTx, when the patient is hemodynamically stable, TPN protocol is continued in the same way as before the transplantation in the ITU. In cases where no rejection is reported in biopsy, oral nutrition is started for the patient. After the start of oral nutrition, the amount of TPN is decreased gradually, and when the patient can tolerate 70% of their daily calorie requirements through enteral feeding, TPN is stopped.

#### Immunosuppressive Therapy After Intestinal Transplantation or Multivisceral Transplantation

In patients with ITx, induction regimen consisted of 1 g methylprednisolone alongside 1 mg/kg thymoglobulin and 500 mg rituximab. Methylprednisolone and thymoglobulin (similar dose) are continued until the fourth day after transplantation, and on the fifth day, a single dose of rituximab is given (similar dose) again. Twenty-four hours after transplantation, tacrolimus is initiated, and doses are adjusted to keep levels between 15 and 20 ng/mL in the first month after ITx. On the fifth day after transplantation, prednisolone 25 mg/day along with mycophenolate mofetil (1 g twice daily up to 3 g/day) and everolimus (serum level of 5-8 ng/mL) is started.

Immunosuppressive regimen among patients with MVT is similar to that of patients receiving an isolated IT with the exclusion of rituximab. All medication doses are adjusted according to patients’ renal function/glomerular filtration rate (GFR) and also other relevant complications.

#### Antibiotic Regimen After Intestinal Transplantation or Multivisceral Transplantation

All patients receive 1 g/q12h vancomycin with 4.5 g/q6h piperacillin-tazobactam, which is continued until the third day after transplantation. As a prophylactic treatment for fungal infections, all patients receive caspofungin (70 mg stat and 50 mg/daily) until oral feeding is started. After oral feeding is started, fluconazole (50 g BID) is started and continued for a duration of 30 days.

For the prevention of pneumocystis pneumonia, all patients receive trimethoprim/sulfamethoxazole 400/80 mg/daily for a period of 1 year. In our center, a prophylactic strategy is used for cytomegalovirus (CMV). Accordingly, all patients receive 450 mg twice a day (BID) valganciclovir for 6 months, and also, they are screened for CMV and Epstein–Barr virus on a weekly manner via polymerase chain reaction, and in cases when a patient becomes symptomatic for infections and/or lab tests are in favor of viral infections, they are visited and followed by an infectious specialist.

#### Intestinal Biopsy for Management of Rejection

During the first month after transplantation, an intestinal biopsy is obtained 2 times every week from each patient by a gastroenterologist. The sample is assessed with regard to the condition of graft and CMV by a pathologist. After the first month, biopsy is taken weekly, and after 2 months, biopsy is obtained every 2 weeks up to the third month, following which biopsy is taken monthly up to 1 year.

In cases of mild to moderate rejection, patients receive 20 mg/kg methylprednisolone for 3 days, and the dose of tacrolimus is increased by 50%.

In cases of severe rejection, patients receive 1000 mg methylprednisolone for 3 days and the dose of tacrolimus, mycophenolic acid, and everolimus is increased by 50% up to the eligible serum level (tacrolimus level up to 13-15 ng/dL). In cases where C4D is positive, patients receive Intravenous Immunoglobulin Therapy (IVIg) with plasmapheresis (with replacement of volume of plasmapheresis with albumin) for 6 sessions.

In cases where biopsy samples are positive for CMV, ganciclovir 5 mg/kg q12h is given to patients for 2 weeks and the dose of immunosuppressive medications is decreased by 20%. After 2 weeks, oral valganciclovir 900 mg BID is given to patients for 6 months.

### Ethical Consideration

All patients gave their written and informed consent to enter the study. All study protocols were in accordance with the guidelines stated in the Declaration of Helsinki and the Declaration of Istanbul for organ transplantation. The study protocol was approved by the Institutional Review Board.

### Statistical Analysis

Data are analyzed using the Statistical Package for the Social Sciences software for Windows, version 25 (IBM Corp.; Armonk, NY, USA). Quantitative data are reported as means and standard deviation (SD) and median and interquartile range (IQR).

## Results

### Patient Description

After the establishment of GI continuity, in patients with ultra-short bowels (less than 20 cm), a gastrostomy tube was inserted as a vent for preoperative preparation prior to transplantation (patient numbers 1 and 2). Patient numbers 3 and 7 were candidates for ITx because they had previously undergone 3 operations due to motility disorders and were still PN dependent. In patient number 6, considering that the remaining bowel length was 40 cm and distended, serial transverse enteroplasty (STEP) procedure was done; however, in the following 1 year period, the patient was still PN dependent and therefore the patient became a candidate for ITx.

Patient number 4 developed bowel gangrene following laparoscopic colectomy and portal thrombosis and had isolated ITx. Similarly, patient number 5 developed bowel gangrene and afterward developed liver failure due to TPN. In patient number 8, the patient developed non-cirrhotic portal thrombosis and multiple episodes of bleeding. Patient number 9 had IF due to a well-differentiated neuroendocrine tumor with mesenteric involvement and a liver mass.

As a result, these 3 patients (5, 8, and 9) became candidates for MVTx (Table 1).

### Clinical Data

The median (IQR) age of patients was 35 (29.5, 48) years (mean [SD] of 37.5 ± 12.5 years). The majority of patients were females (7/9). The median (IQR) number of days to receive TPN was 62 (29.5, 145) days.

The median (IQR) waiting time for patients from diagnosis to transplantation was 79 (34, 164) days. Three patients had multivisceral organ transplantations.

Baseline and preoperative clinical characteristics of patients are shown in Table 1.

Mean blood loss during MVTx and isolated ITx was 3130 cc and 433 cc, respectively. Intraoperative management and clinical characteristics of patients are shown in Table 2.

**Figure 1.**
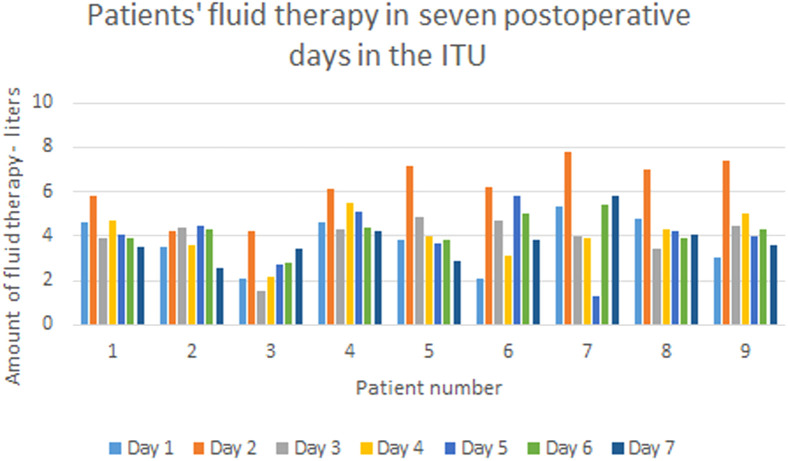
This figure shows a 7-day course of fluid therapy during the postoperative period.

Our 7-day survey of the amount of fluid therapy after transplantation revealed that the greatest need for fluid therapy was seen on the second postoperative day (Figure 1).

The median (IQR) duration of ITU stay was 18 (13.5, 20) days. After transplantation, 2 patients showed a total of 3 episodes of severe rejection, one of which was antibody-mediated.

Patients were followed up to January 2021 (range, 22 to 609 days), during which, 5 patients (55%) died. The 1-year survival was 66.6%, and the estimated 2-year survival was 44.5% in our study population. The median (IQR) time to death was 157 (26.5, 382) days.

The most common cause of death was sepsis in our series (3/5). One patient developed bloodstream infection caused by *Klebsiella*, one patient developed sepsis by methicillin-resistant coagulase-negative *Staphylococci*, and one patient developed sepsis caused by a combination of vancomycin-resistant enterococcus and *Candida* non-*albicans*. One patient died due to opium overdose, and the cause of death was unknown in 1 patient as the patient died in another center and the medical records were not accessible (Table 3).

## Discussion

Intestinal transplantation is one of the rarest types of organ transplantations performed.^9^ To this date, indications of ITx have been established among countries with facilities such as HPN and medications including enterohormones (such as Glucagon-like peptide 2 [GLP-2]).^10-13^ Despite the lack of these facilities and medications, Iran is among the few countries which perform this surgery with locally modified guidelines.^4^ These strategies include establishing an IRU with a multidisciplinary team as a referral center for patients with IF, minimizing complication of TPN by training personnel and patients, and performing AGIR surgeries such as STEP and valve reconstruction to obtain GI autonomy. Considering that long hospitalizations for TPN will result in high infection rates, patients with failed AGIR and ­medical management will be prioritized on the ITx ­waiting list.

The majority of our donors are young and transplantation waiting time is short due to high rates of deadly traffic accidents in Iran. Moreover, considering existing limitations, long durations of PN are not given to our patients, and as a result, complications such as IF-associated liver failure did not occur and combined liver–intestinal transplantation was not performed among our 9 reported patients. Accordingly, all of our transplantations were either isolated ITx or MVTx for causes such as extensive porto-mesenteric thrombosis.

The non-availability of HPN has also affected our indications for ITx. Accordingly, unlike many centers in the world that include development of complications due to HPN as part of their indications for ITx, all of our patients receive a combination of in-hospital TPN and AGIR, after which they are transplanted at the earliest time possible.

Our waiting list is relatively short considering that our number of cases in need of ITx is low and our availability of organs for transplantation is proportionately high.

**Figure 2.**
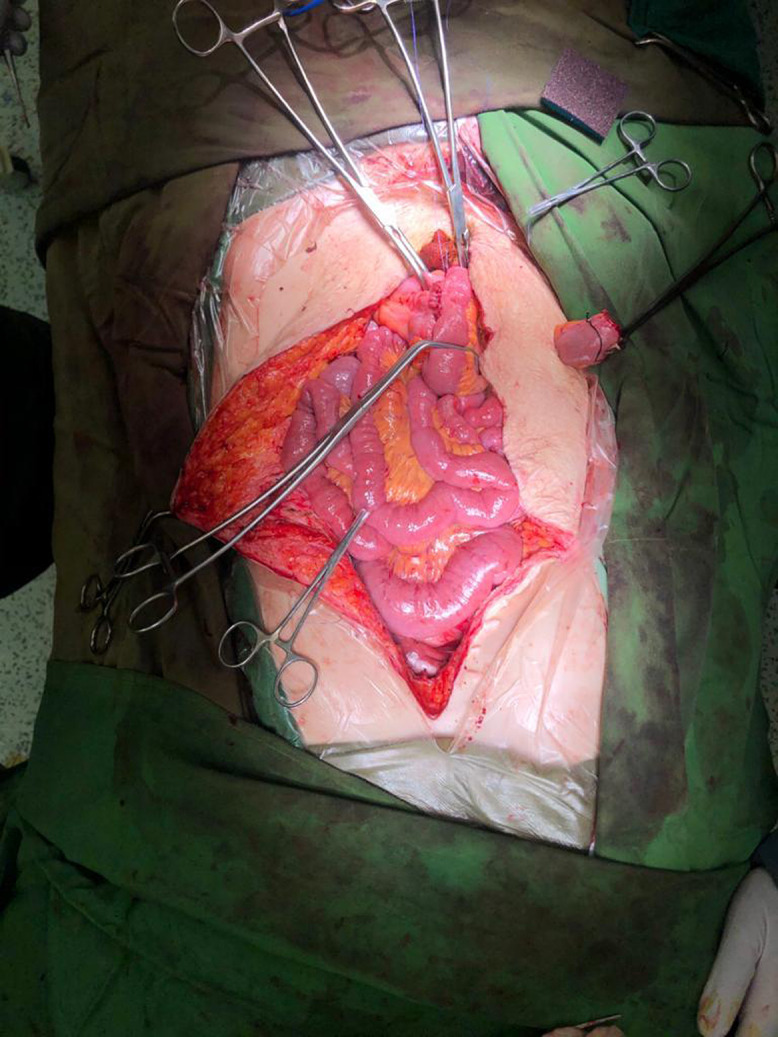
The image shows the transplanted intestine after reperfusion.

Perioperative fluid therapy is a challenging issue for the anesthesiologist during ITx, as excretion of fluids from the vessels can result in intestinal edema; furthermore, assaults from ischemic reperfusion injury can also play a role in the development of intestinal edema. Accordingly, fluid therapy in these patients required reasonable and meticulous monitoring and over-hydration should be avoided. We used a Hartmann’s solution with low-dose albumin infusion to maintain the CVP between 5 and 8 cmH_2_O. When the patient had a variation of more than 15% in their pulse pressure, which was not corrected to under 10% using a challenging fluid therapy of 200 cc fluid, in order to establish hemodynamic stabilization, inotrope medications were used. Considering that anastomosis of the vessels is susceptible in these patients, before reperfusion of the organ using rotational thromboelastography, hypocoagulability state had to be diagnosed and treated, and administration of blood products should be done precisely.^14^ Considering that reperfusion syndromes occur in 4% of ITx, in order to prevent this, serum potassium levels of patients should be maintained lower and equal to 4 mEq/L and any existing acidosis should be corrected to decrease cardiac complication of this syndrome (Figure 2).^15^

Although we have been able to minimize our number of patients in need of ITx using the specific and personalized treatment protocol which include a combination of AGIRs and in-hospital TPN, from another perspective, we expect to have higher mortality rates compared to other centers in the world that perform ITxs that have facilities for HPN, as our patients are transplanted during hospital admission (due to the unavailability of HPN). In a study by Abu-Elmagd et al.^16^ that evaluated the outcomes of intestinal and mutivisceral transplantations in one of the largest intestinal transplantation centers in the United States, among 376 patients from 1990 to 2006, authors reported a 5-year survival of 60% among patients. Although it should be noted that our population only included adult recipients of ITx. Adults are reported to have lower survival rates compared to pediatric patients who undergo ITx.^17^ Moreover, the 2018 annual Organ Procurement and Transplantation report found the 1-year and 5-year survival of adult recipients of ITx to be 66.7% (vs 66.6% in our study) and 49.1%, respectively, which was similar to that of our study.^17^

Among patients with extensive porto-mesenteric thrombosis and slow-growing masses, our indications for transplantation are similar to that of other countries. Patients with motility disorders will become candidates for transplantation after initial decompression surgery.

By establishing a detailed treatment protocol for patients with IF, we have been able to obtain fair postoperative outcomes.

This study was not without limitations. First was the relatively short follow-up as our IRU is newly established and long-term follow-ups will be reported in future studies. Intestinal transplantation and AGIRs require experienced and trained surgeons, and establishing an IRU requires precise coordination between different specialists.

## Conclusion

Although the overall survival of ITx remains to be low compared with other organ transplantations, based on the results of our study, acceptable outcomes can be obtained with ITx in countries without HPN by the application of specific treatment protocols.

From one aspect, the lack of HPN in our country may advocate earlier ITx compared to countries that have facilities for HPN; however, from another aspect, before patients are referred to our center, they usually receive long-term PN and develop complications related to PN due to a lack in experience with PN in many other centers and due to the fact that many centers and professionals are unaware of the practice of ITx in our country (considering that we are the only center in our region that ITx is done), this further delays the early referral of patients to our center.

## Figures and Tables

**Table 1.** Baseline Characteristics and Preoperative Evaluations Among Patients with Intestinal Transplantations

Variables	Patients								
1	2	3	4	5	6	7	8	9
Age (years)	59	29	32	18	35	52	30	39	44
Sex	F	F	F	M	F	M	F	F	F
BMI (kg/m^2^)	17.5	18.7	21.4	13.9	17.3	17.3	16	14.5	19.1
Blood group	A+	O+	A+	O+	A−	A+	B+	A+	A+
Underlying disease	HTN, DM, dyslipidemia, IHD, high liver enzymes	Opium addict	-	-	Liver failure	DVT, heart failure, CVA, seizure	-	Opium addict, COPD, DM, chronic PVT	NET, DM
Waiting time for transplantation (days)	360	146	106	34	79	183	34	25	62
Length of TPN (days)	360	48	106	22	110	180	35	24	62
Type of surgery	IITx	IITx	IITx	IITx	MVTx	IITx	IITx	MVTx	MVTx
Preoperative evaluation									
Renal function	Normal	Normal	Normal	Normal	Normal	Normal	Normal	Normal	Normal
Cardiac function	IHD	Normal	Normal	Normal	Normal	HF	Normal	Normal	Normal
Pulmonary function	Normal	Normal	Normal	Normal	Normal	Normal	Normal	COPD	Normal
Liver function	High enzymes	Normal	Normal	Normal	High enzymes	Normal	Normal	High enzymes	Normal
Coagulopathy	Normal	Normal	Normal	INR>1.5	Normal	INR >1.5 & low plt	Normal	Low plt	Normal
Viral marker	Neg	Neg	Neg	Neg	Neg	Neg	Neg	Neg	Neg
FBS	>120	Normal	Normal	Normal	Normal	Normal	Normal	>120	>120
Albumin (g/dL)	2.5	4.3	3.5	2.1	3.3	2	4.1	4.5	3.1
Total bilirubin (mg/dL)	1.22	0.66	0.24	0.65	3.9	0.74	0.39	6.88	0.58
Direct bilirubin (mg/dL)	0.38	0.22	0.11	0.37	2.25	0.16	0.09	3.24	0.25
Creatinine (mg/dL)	0.5	0.7	0.6	0.8	0.5	0.9	0.9	0.7	0.5
Vitamin D level	28.7	106.	9.5	5.4	17.9	10.9	17.6	20.9	-
Calcium*	Low	Nl	Nl	Low	Low	Low	Low	Nl	Nl
Phosphorus*	High	Low	Nl	Nl	Nl	Low	Nl	Low	Nl
Magnesium*	Low	Low	Low	Low	Low	Low	Nl	Nl	Low

IF, intestinal failure; TPN, total parenteral nutrition; FBS, fasting blood sugar; HTN, hypertension; DM, diabetes mellitus; IHD, ischemic heart disease; INR, international normalized ratio; DVT, deep vein thrombosis; CVA, cerebrovascular event; COPD, chronic obstructive pulmonary disease; NET, neuroendocrine tumor; Plt, platelet; PVT, portal vein thrombosis; IITx, isolated intestinal transplantation; MVTx, multivisceral transplantation; Neg, negative; Nl, normal.*The normal laboratory range for calcium, phosphorus, and magnesium (for males and females) were 8.6-10.3 mg/dL, 2.-4.5mg/dL, and 1.9-2.5 (for males) and 1.8-2.6 mg/dL (for females).

**Table 2.** Intraoperative Assessment and Clinical Characteristics Among Patients with Intestinal Transplantations

Variables	Patients								
Fluid therapy	1	2	3	4	5	6	7	8	9‡
Crystalloid (cc)	3000	2500	2000	2000	5500	500	3000	2500	3000
Colloid (cc)*	500	-	500	1000	-	1000	2000	3000	500
Inotropes	−	+	−	−	+	+	−	−	+
Albumin (g)	30	-	-	20	55	5	-	25	30
U/O (cc/kg/h)	3.7	6.9	3.6	2.8	4.5	3.6	2.7	2.7	4.3
Crystalloid index (cc/kg/h)	11.1	11.5	8	8.8	15.7	1.8	13.8	6.9	13
Colloid index (cc/kg/h)	1.85	-	2.02	4	-	3.6	9.2	8.3	2.1
Surgery time (hours)†	6	4.30	4.30	5	7	5.30	6	6.30	5
Blood loss (cc)	450	200	500	500	2700	150	800	4700	2000
Packed cell (bags)	1	-	-	2	5	-	2	7	5
ROTEM	-	Impaired	-	Normal	Normal	Normal	-	Impaired	Normal
FFP (bags)	-	3	-	-	-	-	-	4	-
Cryoprecipitate	-	-	-	-	-	-	-	5	-
Hemoglobin before surgery (g/dL)	10.7	13.5	11	11.	9.3	13.3	12.1	9.4	11.9
Hemoglobin start of transplantation	10.1	12.4	11.9	9.5	10.6	8.9	13.2	9	7.5
Hemoglobin end of transplantation (g/dL)	11.3	12.5	11.1	11.2	9.7	10	10.4	11.8	10.4
Plt start of transplantation (×10^3^)	150	508	274	363	527	82	220	92	171
INR start of transplantation	1	1	1	1.94	1.12	1.6	1.3	1.35	1
Intraoperative medication use									
Vancomycin (g)	1	1	1	1	0.5	1	1	1	1
Duration of use (days)	2	2	2	2	2	2	2	2	2
Piperaciline (g)	4.5	4.5	4.5	4.5	4.5	4.5	4.5	4.5	4.5
Duration of use (days)	2	2	2	2	2	2	2	2	2
Methyl prednisolone (g)	1	1	1	1	1	1	1	1	1
Duration of use (days)	4	4	4	4	4	4	4	4	4
Rituximab (mg)	500	500	500	500	-	500	500	-	-
Thymoglobulin (g)	50	50	50	50	50	75	50	75	75
Duration of use (days)	4	4	4	4	4	4	4	4	4
Acid–base treatment									
Na-HCO_3_ (7.5%)^¶^	150	150	150	-	350	150	-	200	300
KCl (15%)	-	-	-	-	10	-	20	5	-
U/O, urine output; ROTEM, rotational thromboelastography; FFP, fresh frozen plasma *The colloid used was gelfusin. †Surgery time was considered without time of harvest. ‡This patient also received 300 µg octreotide due to underlying NET. ¶It seems that patients with more blood transfusion required more sodium bicarbonate.									

**Table 3.** Postoperative Assessment and Clinical Characteristics Among Patients with Intestinal Transplantations.

Variables	Patients								
1*	2*	3	4	5	6	7	8	9
Duration of intubation (days)	2	1	1	1	1	2	1	3	1
Duration of admission in ITU (days)	27	12	15	8	20	17	20	18	20
Mean received fluid therapy (cc/kg/h)	4.39	3.92	2.73	4.90	4.35	4.40	4.81	4.57	4.64
Fluid therapy on day 1 (cc/kg/h)	4.67	3.5	2.1	4.6	3.8	2.1	5.3	4.8	3.7
Fluid therapy on day 2 (cc/kg/h)	5.8	4.2	4.2	6.1	7.2	6.2	7.8	7	7.4
Fluid therapy on day 7 (cc/kg/h)	3.5	2.6	3.4	4.2	2.9	3.8	5.8	4.1	3.6
Mean U/O	2.4	3.1	2.4	3.5	2.8	3.1	4.6	3.3	3
Mean albumin (g/dL)	2.7	3	2.8	2.2	2.4	2.4	2.4	2.2	2.4
ABG indices									
After reperfusion									
PH (mm Hg)	7.43	7.46	7.26	7.40	7.34	7.32	7.47	7.39	7.37
PCO_2_ (mm Hg)	29	33	35	32	36	31	33	38	39
HCO_3_ (mEq/L)	19.2	23.5	16.3	20.4	19.2	15.8	24	22.6	22.6
BE (mEq/L)	−4	−0.2	−10.3	−2.3	−6.4	−8.3	1	−1.7	−3.3
Day 1 of ITU admission									
PH	7.18	7.48	7.30	.31	7.31	7.30	7.35	7.21	7.34
HCO_3_	7.5	22	18	24.3	14	12.9	16	18.2	1.5
BE	−18	−3	−9	−1.9	−12.3	−13.4	−9.6	−9.6	−8.3
Final day of admission									
PH	7.32	7.38	7.23	7.41	6.95	7.44	7.44	7.42	7.46
HCO_3_	14.6	13.4	18.3	24.5	11.7	23.2	20	28.4	27.8
BE	−11.4	−11.7	−9.2	−0.2	−20.2	−0.7	−4.1	4.1	4
Treatment during admission									
Dialysis	-	-	-	-	-	-	-	-	-
Blood transfusion	+	-	+	-	+	-	+	+	+
Inotropes	-	-	-	-	+	+	+	-	+
Diuretic medication	+	-	-	+	+	-	+	+	+
Nutrients on last day of ITU									
Calcium	Low	Low	Nl	Low	Low	Low	Nl	Low	Low
Phosphorus	Low	Low	Nl	Low	Nl	Nl	Nl	Nl	Nl
Magnesium	Low	Low	Low	Low	Nl	Low	Nl	Low	Nl
Post-transplantation complication		-							
Mild to moderate rejection (no.)	2	0	1	2	0	1	0	1	0
Severe rejection	0	0	1	2	0	0	0	0	0
Antibody-mediated rejection (no.)	0	0	0	1	0	0	0	0	0
Infection (no.)	SSI + UTI + BSI	0	SSI	BSI + UTI	BSI + pneumonia	BSI	BSI	BSI	HSV esophagitis
CMV infection	+	-	-	+	-	-	-	-	-
Other complications	Convulsion	-	-	Pneumonia	-	Pseudomembranous colitis	-	GOO, pancreatitis	Fistula at site of surgery
Prognosis	Died	Died	Alive	Alive	Died	Died	Alive	Died	Alive
Cause of death	Sepsis	Opium overdose	-	-	Sepsis	Sepsis	-	Unknown	-
Survival duration (days)	157	31	609	455	22	383	402	381	291
U/O, urine output; ABG, arterial blood gas; BE, base excess; pH, acid- base level; CMV, cytomegalovirus; ITU, intensive transplantation unit; SSI, surgical site infection UTI, urinary tract infection; BSI, bloodstream infection; HSV, herpes simplex virus; GOO, gastric outlet obstruction; Nl, normal. *Patient nos. 1 and 2 had a jejunostomy tube in the ITU. In patient no. 1, normal saline fluid (0.9%) was used to replace the excreted fluid from the jejunostomy tube, however, due to the subsequent severe acidosis, Ringer’s lactate was used as a replacement fluid for other patients.									

**Table:** 

**Indications for intestinal transplantation in the Shiraz Transplant Center.**
1. Short bowel syndrome unable to obtain intestinal autonomy with autologus gastrointestinal reconstruction
2. Motility disorders unable to become off parenteral nutrition despite decompressive surgery
3. Extensive portomesentric thrombosis with liver failure
4. Slow growing tumors occupying intestinal mesentry (such as well differentiated neuroendocrine tumors, desmoid tumors and gastrointestinal stromal tumors)
5. Intestinal failure associated with liver failure

## Supplementary 2. Anesthesia Intraoperative Care and Management

### Monitoring

Basic monitoring is used for all the patients which includes ECG, non-invasive blood pressure (NIBP), pulse oximetry, temperature monitoring and capnography.

After standard induction of anesthesia, an arterial line is obtained from the non-dominant upper extremity and a central venous catheter is set in the internal jugular vein under the guide of ultrasonography. Pulse contour cardiac output(PiCCO) is used to monitor cardiac output, systemic vascular resistance, and pulse pressure variation, and the fluid therapy is partially adjusted based on these indices. An arterial line is set to monitor the blood pressure (BP) and arterial blood gas (ABG) during surgery. Through the central venous catheter, patients’ central vein pressure (CVP) and goal directed fluid therapy is monitored and managed. In order to minimize hypothermia during surgery, an air force blanket is used.

### Fluid therapy

Fluid management protocol is based on our institutional experiences with organ transplantation. The basis of fluid therapy among isolated ITx or MVTx recipients in our center is goal directed fluid therapy. Accordingly, fluid therapy is managed based on patients’ CVP, heart rate and BP and is done using a combination of crystalloids and colloid solutions.

Fluid therapy initially starts with administration of 7 cc/kg isotonic crystalloid and during surgery, the CVP is maintained between 5-8 cmH_2_O. Minimizing fluid therapy is crucial for preventing intestinal edema and consequent weakening of anastomosis and its deleterious effects on surgery outcomes.^1^ Therefore, when fluid therapy is not sufficient enough to maintain a mean arterial pressure (MAP) of ≥ 60 mm Hg, vasopressors and inotropes are administered. To optimize the condition of the liver and to minimize its congestion, the CVP is maintained at the lower limit of the aforementioned range.^2^

In all patients who either have serum albumin levels of <3 g/dL or undergone MVTx and have received liver transplantations, 10 gr of albumin is given with every 1000 cc crystalloid solution.

### Acid-base balance

For all patients, arterial blood gas (ABG) is measured at three intervals: first at the beginning of the surgery; second after organ extraction and before the initiation of the new anastomosis in order to correct the acid-base condition; and third, one hour after finishing all the anastomosis, removing of the clamps, and starting organs’ perfusion.

In cases in which additional electrolyte imbalance evaluation is needed or acute hemodynamic changes occur, additional ABG is measured at any of the aforementioned stages and any existing imbalance is corrected accordingly. Using the second ABG and in order to prevent probable acidosis caused by reperfusion syndrome, base excess (BE) is corrected to zero using sodium bicarbonate. Moreover, to prevent hyperkalemia caused by either reperfusion syndrome or probable blood transfusion, serum potassium levels are corrected at a higher threshold and is corrected at k<2.5 mmol/L. Potassium levels between 2.5 and 3.5 mmol/L is merely corrected in cases of cardiac arrhythmia or in cases in which patient’s hemodynamic changes is unresponsive to neither fluid therapy nor use of inotropes. After declamping and finalization of anastomosis, all potassium levels below 3.5 mmol/L are corrected.

### Blood loss and coagulation

The goal of bleeding control among these patients is to maintain hemoglobin levels at 9-10 g/L^3^ and to prevent any blood oozing from the site of surgery caused by probable coagulopathy. Therefore, patients’ hemoglobin levels are managed by administration of packed cells and coagulopathy is controlled purposefully after rotational thromboelastometry (ROTEM) is applied. The criteria for ROTEM application is as follows: transfusion of more than 4 packed cells (considered as massive transfusion),^4^ INR>1.5, or blood oozing from site of surgery. In order to evaluate the quality of bleeding control and coagulopathy, hemoglobin is checked at the end of surgery. In cases for which fresh frozen plasma (FFP) is needed, 15 cc/kg is transfused and the dosage of cryoprecipitate is 5 units in each transfusion for selected patients.^5^

This method has been successfully applied for minimizing the use of blood products considering the limited resources available to us.

### Total parenteral nutrition protocol

In patients with sepsis or septic shock, TEE in the early phase of sepsis is decreased to 70% of the initially measured TEE. In patients with a BMI> 30 kg/m^2^, TEE is decreased to 70% and a high protein and low calorie TPN regimen is considered for these patients.

All patients receiving TPN are visited daily by the clinical pharmacist and if complications such as refeeding syndrome, electrolyte imbalance, volume overload, catheter infections or etc. occur, they are treated accordingly to scientific guidelines.

In cases when parenteral nutrition liver associated disease (PNLAD) occurs, non-protein energy expenditure decreased to 70% and the lipid source is changed from soy bean based to Smoflipid ®(SMOF). Moreover, continuous infusion is changed to cyclic infusion according to the previous studies.^6,7^
